# Radical resection of an initially unresectable intrahepatic cholangiocarcinoma after chemotherapy with using gemcitabine, cisplatin, and S-1: report of a case

**DOI:** 10.1186/s40792-019-0656-z

**Published:** 2019-06-24

**Authors:** Masashi Tsunematsu, Koichiro Haruki, Taro Sakamoto, Tadashi Uwagawa, Hiroaki Shiba, Katsuhiko Yanaga

**Affiliations:** Department of Surgery, The Jikei University School of Medicine, 3-25-8, Nishi-Shinbashi, Minato-ku, Tokyo 105-8461 Japan

**Keywords:** Metastatic biliary tract cancer, Gemcitabine, Cisplatin, S-1, Conversion surgery

## Abstract

**Background:**

Metastatic biliary tract cancer (BTC) has poor prognosis. Recently, patients with metastatic BTC who respond well to systemic chemotherapy can be treated by radical resection or “conversion surgery.”

**Case presentation:**

A 67-year-old male patient was diagnosed with intrahepatic cholangiocarcinoma with para-aortic metastases [cT2N1M1, stage IVB]. He was enrolled in our phase II study for unresectable BTC consisting of cisplatin (25 mg/m^2^ i.v. for 30 min) followed by gemcitabine (1000 mg/m^2^ i.v. for 30 min) on days 1 and 8 and oral S-1 on alternate days. After 8 courses of this regimen, marked regression of para-aortic lymph metastases was achieved, and we performed extended left hepatic lobectomy with the caudate lobe, concomitant portal vein resection, and lymph node dissection including the para-aortic region. The patient made a satisfactory recovery and was discharged on postoperative day 25. Histopathological examination revealed more than 50% of the tumor area replaced with fibrosis, and curative resection was achieved (ypT2N1M1, stage IVB, Evans criteria IIb). The patient received adjuvant chemotherapy using S-1 for 12 months and remains well with no evidence of tumor recurrence as of 48 months after surgery.

**Conclusions:**

We herein report a successfully treated case of advanced BTC with para-aortic lymph node metastases by conversion surgery after combination chemotherapy using gemcitabine, cisplatin, and S-1.

## Background

Patients with unresectable biliary tract cancer (BTC) with locally advanced or metastatic lesions have poor prognosis [1], for whom systemic chemotherapy is the standard treatment for unresectable BTC [2]. Gemcitabine plus cisplatin combination chemotherapy (GC) are currently recognized as standard treatments for unresectable BTC, but a median overall survival has been reported as only 11.7 months in the ABC-02 study [3]. Therefore, a new treatment strategy for unresectable BTC needs to be developed. S-1, which is an oral fluoropyrimidine, is widely used for BTC in Japan. We started a phase II study for unresectable BTC consisting of cisplatin (25 mg/m^2^ i.v. for 30 min) followed by gemcitabine (1000 mg/m^2^ i.v. for 30 min) on days 1 and 8 and oral S-1 on alternate days (GCS) since 2015.

Conversion surgery is a possible treatment strategy for the potential cure of primarily unresectable hepatobiliary pancreatic cancer. We herein report a successfully treated case of primary unresectable intrahepatic cholangiocarcinoma due to para-aortic lymph node metastases by conversion surgery after GCS.

## Case presentation

**Fig. 1:**
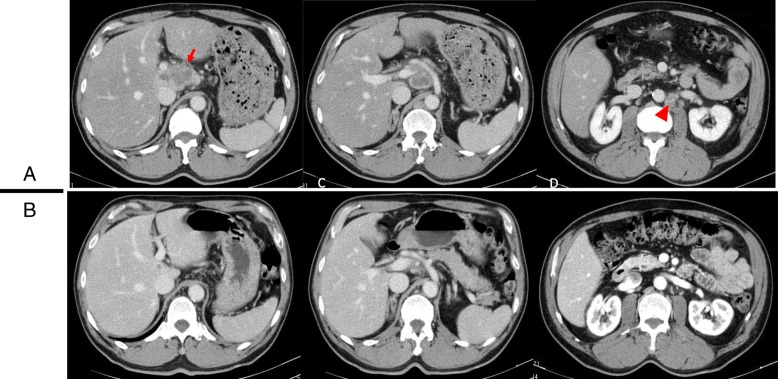
Pre- and post-chemotherapy enhanced computed tomography scan findings. **a** A hypovascular mass in the segment 1 (arrow), which invaded to the portal vein and showed multiple lymph node metastases including para-aortic lymph node (arrowheads). **b** Following chemotherapy, the complete response of para-aortic lymph node metastases was achieved

**Fig. 2:**
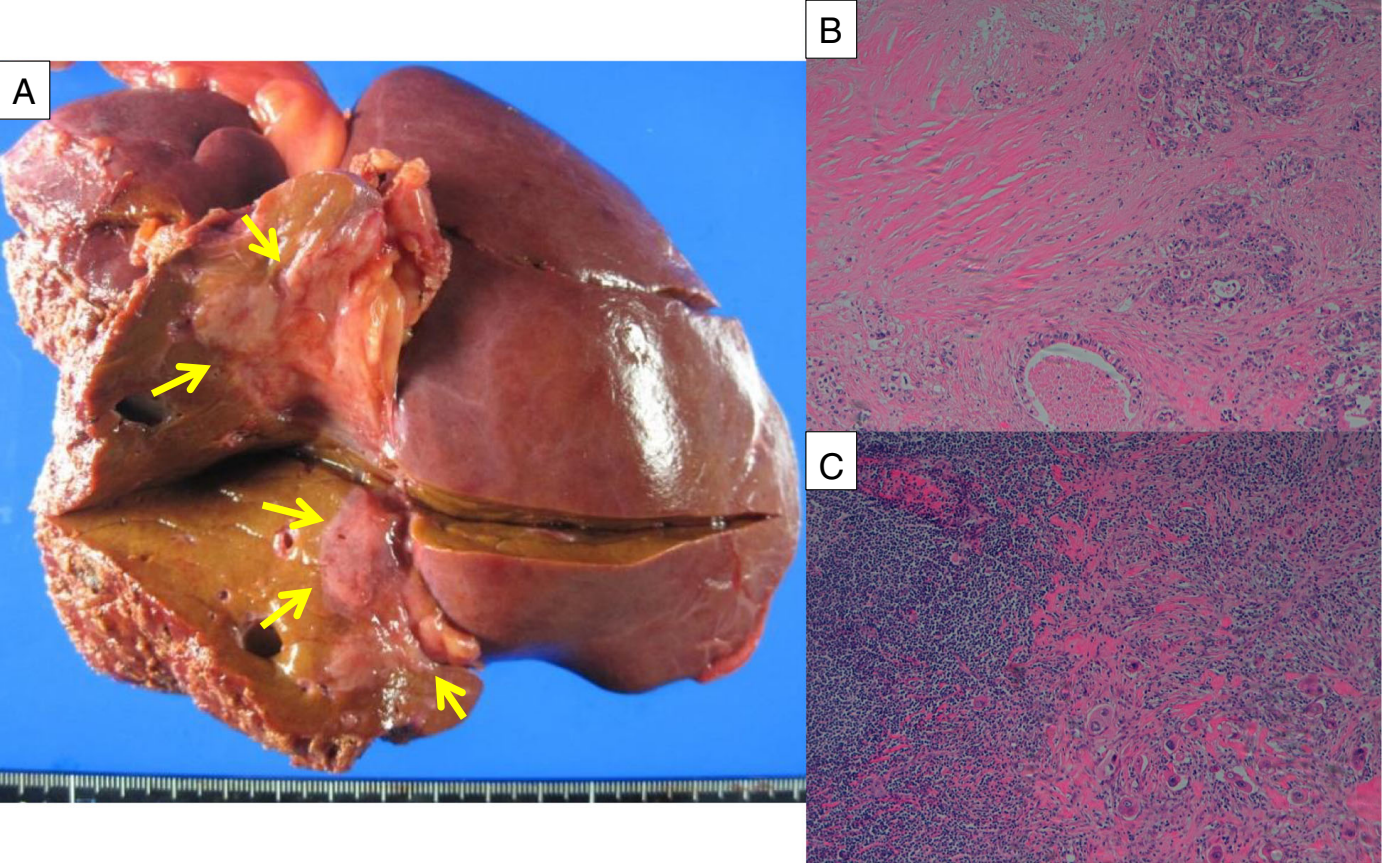
**a** Surgical specimen. **b** Microscopic findings of surgical specimen stained with hematoxylin and eosin, demonstrating more than 50% of the tumor area replaced with fibrosis (Evans criteria IIb). **c** Microscopic finding of the lymph node invasion

A 67-year-old male (height 163 cm, body weight 70.0 kg) was referred to our hospital for evaluation of an abdominal tumor which was pointed by abdominal ultrasonography during his health checkup. An enhanced computed tomography scan revealed a hypovascular tumor in the segment 1 of the liver (6 cm diameter), which invaded to the portal vein and showed multiple lymph node metastases including para-aortic ones (Fig. 1a). Brushing cytology of the bile duct yielded a diagnosis of a moderately differentiated adenocarcinoma. The clinical diagnosis was unresectable advanced intrahepatic cholangiocarcinoma with para-aortic lymph node metastases (cT2N1M1, stage IVB according to the Japanese classification system, the 6th edition). The serum CA19-9 level was 2323 U/ml. The patient received systemic chemotherapy [gemcitabine (1000 mg/m^2^) and cisplatin (25 mg/m^2^) infused on days 1 and 8 and S-1 administered orally (120 mg/day) on alternate days]. This regimen was repeated at 21-day intervals. After 8 courses of GCS, the primary tumor and para-aortic lymph nodes showed remarkable regression (Fig. 1b). The tumor size was 3 cm in diameter. The primary tumor was regarded as partial response, while the para-aortic metastases were regarded as complete response. The synthesis was partial response according to the RECIST 1.1 standard. The serum CA19-9 level decreased to 11 U/ml. The relative dose intensities for gemcitabine, cisplatin, and S-1 were 93.5%, 87.5%, and 68.8%. As adverse effects were limited to grade 2 creatinine elevation (Common Terminology Criteria for Adverse Events version 4.0), the patient underwent conversion surgery. Intraoperative frozen section showed no evidence of para-aortic lymph node metastases. Therefore, the patient underwent extended left hepatic lobectomy with the caudate lobe, concomitant portal vein resection, and lymph node dissection including the para-aortic region. The excised specimen showed a solid tumor with a diameter of 26 × 17x12 mm in the caudate lobe of the liver (Fig. 2a). Pathological findings revealed more than 50% of the tumor area replaced with diffuse fibrosis (Fig. 2b) and one of the para-aortic lymph nodes had residual tumor cells and fibrosis (Fig. 2c). Curative resection was achieved (ypT2N1M1, stage IVB, Evans criteria IIb). After surgery, the patient developed perforation of a duodenal ulcer and was treated by ultrasonography-guided percutaneous drainage and antibiotics. Thereafter, the patient made a satisfactory recovery, was discharged on postoperative day 25, and received adjuvant chemotherapy with S-1 for 12 months. The patient remains well with no evidence of tumor recurrence as of 48 months after surgery.

## Discussion

Conversion surgery for BTC appears to have potential benefits for patient survival. In the field of colorectal cancer, the resection rates of metastases and prognosis after resection have been associated with the tumor response to chemotherapy [4]. Likewise, the positive correlation seems to exist between the response rate and the resection rate in BTC.

In the ABC-02 study which defined GC as the standard treatment for unresectable and metastatic BTC compared with gemcitabine alone, the response rate was 26.1 vs 15.5% [3]. In the BT-22 study, which evaluated the efficacy and safety of GC compared with gemcitabine alone in Japanese patients with BTC, the overall response rate was 19.5 vs 11.9%, respectively [5]. These studies suggested that GC had a higher response rate than gemcitabine alone. On the other hand, JCOG0805 study, which evaluated the efficacy and safety of gemcitabine plus S-1 combination therapy (GS) in comparison with gemcitabine alone, revealed the response rate of 36.4 vs 17.4% [6]. According to the results, GS requires less burden for patients in terms of antiemetic drugs, time for intravenous injection, nephropathy, and neuropathy. In addition, GS may have a higher response rate than GC. Based on these studies, a randomized phase III trial (JCOG1113 study) is ongoing to confirm the non-inferiority of GS relative to GC, which is the current standard treatment for chemo-naive patients with unresectable or recurrent BTC [7]. Therefore, our group performed a phase I trial of GCS, triplet regimen [8]. As this trial successfully demonstrated the safety and tolerability of GCS, some phase II trial was performed or are ongoing [9–12]. Our GCS regimen (UMIN000016913) is different from the others in that we choose an alternative day administration of S-1 and achieve a higher dose intensity of gemcitabine and cisplatin, by which several cases achieved conversion surgery [13].

There are also several reports on surgeries following chemotherapy for initially unresectable advanced BTC [14, 15]. These patients were good responders to initial systemic chemotherapy. The serum CA 19-9 levels of these cases were within the normal range for several months before conversion surgery. They achieved a partial response or a stable disease with a small number of courses. For such patients, conversion surgery is a suitable approach which improves the poor prognosis of unresectable BTC. However, there is insufficient evidence regarding the optimal regimen and number of courses required for considering conversion surgery. Therefore, the possibility of conversion surgery should be judged by periodic imaging diagnosis and transition of serum tumor marker. At our institute, we evaluate the tumor using enhanced computed tomography scan following every 2 courses.

In the present case, conversion surgery had a major impact on the patient’s survival: a disease-free survival of 48 months after surgery. Further studies are needed to evaluate and determine the optimal regimens, as well as the suitable number of courses for each unresectable lesion.

## Conclusions

We reported a case of successful conversion surgery for intrahepatic cholangiocarcinoma with para-aortic lymph node metastases. Further studies and careful assessment are necessary to discuss whether or not such a conversion surgery can be conducted safely and with certainty and to what extent patient survival is prolonged.

## Data Availability

All data generated or analyzed during this study are included in this published article.
